# Phenotypic heterogeneity in human genetic diseases: ultrasensitivity-mediated threshold effects as a unifying molecular mechanism

**DOI:** 10.1186/s12929-023-00959-7

**Published:** 2023-07-31

**Authors:** Y. Henry Sun, Yueh-Lin Wu, Ben-Yang Liao

**Affiliations:** 1 Institute of Molecular and Genomic Medicine, National Health Research Institute, Zhunan, Miaoli Taiwan; 2 Institute of Molecular Biology, Academia Sinica, Taipei, Taiwan; 3 Division of Nephrology, Department of Internal Medicine, Wei-Gong Memorial Hospital, Miaoli, Taiwan; 4 Division of Nephrology, Department of Internal Medicine, Taipei Medical University Hospital, Taipei, Taiwan; 5 TMU Research Center of Urology and Kidney, Taipei Medical University, Taipei, Taiwan; 6 Division of Nephrology, Department of Internal Medicine, Wan Fang Hospital, Taipei Medical University, Taipei City, Taiwan; 7 Institute of Population Health Sciences, National Health Research Institute, Zhunan, Miaoli Taiwan

**Keywords:** Phenotypic heterogeneity, Penetrance, Expressivity, Pleiotropy, Stochasticity, Threshold, Ultrasensitivity, Ultrasensitive response motif (URM), Network, Edgetic mutation

## Abstract

Phenotypic heterogeneity is very common in genetic systems and in human diseases and has important consequences for disease diagnosis and treatment. In addition to the many genetic and non-genetic (e.g., epigenetic, environmental) factors reported to account for part of the heterogeneity, we stress the importance of stochastic fluctuation and regulatory network topology in contributing to phenotypic heterogeneity. We argue that a threshold effect is a unifying principle to explain the phenomenon; that ultrasensitivity is the molecular mechanism for this threshold effect; and discuss the three conditions for phenotypic heterogeneity to occur. We suggest that threshold effects occur not only at the cellular level, but also at the organ level. We stress the importance of context-dependence and its relationship to pleiotropy and edgetic mutations. Based on this model, we provide practical strategies to study human genetic diseases. By understanding the network mechanism for ultrasensitivity and identifying the critical factor, we may manipulate the weak spot to gently nudge the system from an ultrasensitive state to a stable non-disease state. Our analysis provides a new insight into the prevention and treatment of genetic diseases.

## Background

People do not respond uniformly to medical conditions and genetic perturbations. For example, upon COVID-19 viral infection, not everyone is symptomatic. Only a small proportion of infected individuals become seriously ill. Upon COVID-19 vaccination, not everyone is protected, and only a small proportion of individuals develop severe adverse effects. Individuals carrying the same oncogenic mutation do not all develop a tumor, not every cell develops into a tumor, and the severity and time of onset vary greatly. Moreover, the response to drugs or therapy also varies greatly. Phenotypic heterogeneity in genetic systems and human diseases is very common. The causes may be genetic, non-genetic (e.g., epigenetic, environmental), or stochastic (e.g., personal immune history) [1, 2]. In this review, we will only address those diseases that have an underlying genetic basis, i.e., a necessary condition for expressing the basic phenotype is the presence of a genetic variant. For simplicity, we will focus on phenotypic heterogeneity caused by germline (heritable) mutations, i.e., all cells in the body carry the same primary (driver) mutation. Somatic mosaicism and sporadic cancers will not be discussed. We will use loss-of-function variants as examples to illustrate our points. The same concept can also be applied to gain-of-function and dominant-negative variants. Some famous examples of phenotypic heterogeneity in human genetic disease are cystic fibrosis [3], Huntington’s disease [4], and Marfan syndrome [5].

**Fig. 1:**
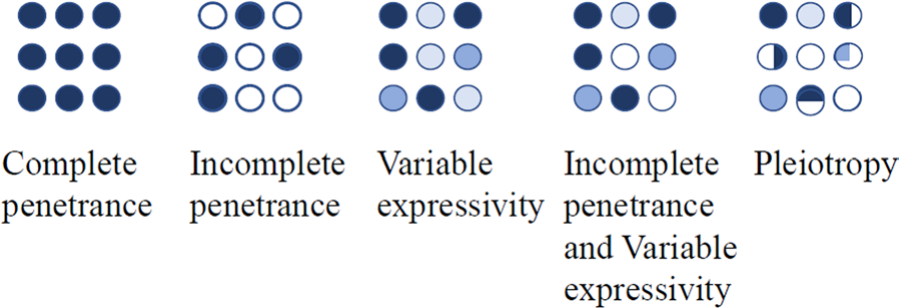
Genotype–phenotype correlations.Penetrance is the percentage of individuals carrying a particular genotype (e.g., a mutation) that exhibits certain detectable phenotypic traits (clinical manifestation). A complete penetrance means that 100% genotype–phenotype correlation. Expressivity is the degree of phenotypic severity in an individual that exhibits detectable mutant phenotype. Pleiotropy means a gene, when mutated, is linked to multiple phenotypic defect, often in multiple tissues or organs

The genotype–phenotype relationship is often described by several terms: penetrance, expressivity and pleiotropy (Fig. 1). Incomplete penetrance and variable expressivity are common in human genetic diseases [6, 7]. Since a gene can have multiple variants with different phenotypic effects, penetrance and expressivity refer to the phenotype associated with a particular genetic variant or allele, not the gene. Phenotypic severity can be different in different affected tissues.

This genotype–phenotype variability is well established in classical genetics. “Partial coupling” was discussed by Bateson in 1907 [8]. Partial penetrance was noted in the first batch of *Drosophila* mutants identified by Thomas Morgan [9] in the *Truncate* mutation causing truncated wing phenotype [10]. Altenburg and Muller [10] proposed in 1920 and demonstrated the existence of multiple modifier genes. Mendelian genetics was initially based on the high penetrance of genetic variants (e.g., Mendel’s pea and Morgan’s *white* mutation in *Drosophila*). It is only in genetically well controlled animals that the phenomenon was first noted.

Penetrance and expressivity are operational definitions as they are determined by the ability to detect the genotype and the phenotype (clinical manifestation). There is variability in phenotype detection, sometimes determined by the clinical criteria used. In some cases, the phenotype may be a continuum but may become a step function based on artificial criteria. For example, the clinical definition for hypertension is an artificial criterion based on population studies, and a cutoff is set on a continuous curve. In other cases, the phenotype may be discrete, e.g., lethality, or appear to be discrete, e.g., tumor. The manifestation of phenotype can also depend on physiological or environmental conditions. Some early developmental defects can be self-corrected; therefore, the phenotype is not detected in later life (e.g., [11]). Conversely, late onset phenotypes are not detected early in life. There is also variation in genotype detection, in that not all people who carry a particular variant are examined. In summary, penetrance and expressivity are quantitative representations of “phenotypic severity” (or, “effect size” in medical usage).

Due to partial penetrance and variable expressivity, patients and carriers of a disease-causing mutation may not be properly diagnosed. This not only underestimates disease prevalence but also reduces the opportunity to provide proper genetic counseling or early treatment. More importantly, we should be asking why some individuals do not develop or have mild disease phenotypes, despite carrying a disease-causing variant. If we are able to better probe the mechanism behind this phenomenon, we may be able to find ways to manipulate the system to reduce or prevent the symptoms. Thus, studying the mechanism of phenotypic heterogeneity may provide an entry point for disease prevention and therapy.

### Numerous causes for phenotypic heterogeneity

If we take the disease-causing mutation as a fixed factor in carriers, variability in phenotypic severity must be due to factors other than the primary (driver) mutation. Phenotypic heterogeneity among different individuals can be due to differences in environmental factors or in genetic background. Examples of environmental factors include nutrition, maternal–fetal interactions, environmental toxic compounds or microbiota [1, 12–16]. The contribution of genetic background has been shown by studies in yeast, *C. elegans*, *Drosophila*, mice and humans [17–26]. Differences in genetic background can be due to the influence of other genes, i.e., modifier genes [27]. Indeed, many genetic screens in model organisms are based on screening for modifiers, either enhancer or suppressor, of existing mutant phenotypes. Modifier genes can be members of the same gene family or genes with redundant functions [28–30], acting in the same functional pathway [31], or involved in protein complex formation [32].

However, there is phenotypic heterogeneity even in isogenic strains of model organisms and in individuals who are genetically “identical”, e.g., in monozygotic twins or cloned cats [33, 34]. There is phenotypic heterogeneity even in a genetically clonal population of single cells (*E. coli*, yeast) grown in the same culture [35, 36]. In KIF11-associated retinopathy, the left eye and right eye of the same individual may have different phenotypic severities [37]. These examples suggest that stochasticity can contribute to phenotypic heterogeneity. Stochasticity originates from fluctuations due to intrinsic non-deterministic phenomena in gene expression or molecular interactions, especially when very small number of molecules are involved. For example, stochasticity can occur in gene expression or in unequal partitioning of molecules during cell divisions [38–41].

Different alleles of a gene may have different penetrance, suggesting that different alleles have distinct probability in exhibiting the phenotype. How is the probability determined in molecular terms? We invoke the threshold concept to explain probability in the next sections. Threshold plus stochasticity are the core components of our model.

### Many gene functions require a threshold level

For most genes, heterozygosity for null mutations does not cause an obvious phenotype, defining them as recessive mutations. However, further reductions in the level/activity may result in a phenotype. Phenotypic severity and gene level/activity are often not linearly proportional and many biological decisions require a threshold level of a key component [42].

For example, spinal muscular atrophy (SMA), a motor neuron degeneration disorder is caused by loss of the SMN1 gene and the phenotypic severity is dependent on the level of SMN2, suggesting a threshold requirement for SMN protein (reviewed by [43]). The genetic compensatory response, where genetic compensation is induced by null mutations (total loss) but not gene knockdowns (partial loss) [44], also suggests a threshold effect in inducing the compensatory response. These examples demonstrate that many gene functions require a threshold level.

Thresholds can vary in different tissues. For example, different tissues have different sensitivities to the deficits in mitochondrial oxidative phosphorylation (OXPHOS) complexes [45]. The m.3243A > G variant in the mitochondrial encoded tRNA^Leu[UUR]^ gene is associated with incredibly diverse disease conditions and has very heterogeneous clinical phenotypes that are dependent on the mtDNA heteroplasmy level, the ratio of mutant versus wildtype mtDNA within a cell [46, 47]*.* Modest changes in the level of a variant can lead to abrupt changes of transcriptional profiles and cellular physiology [48–50]. If different tissues/organs have different tRNA^Leu[UUR]^ expression levels and a different reserve levels of wild type molecules, then their thresholds would be different.

### Threshold effect as a unifying principle for heterogeneity

**Fig. 2:**
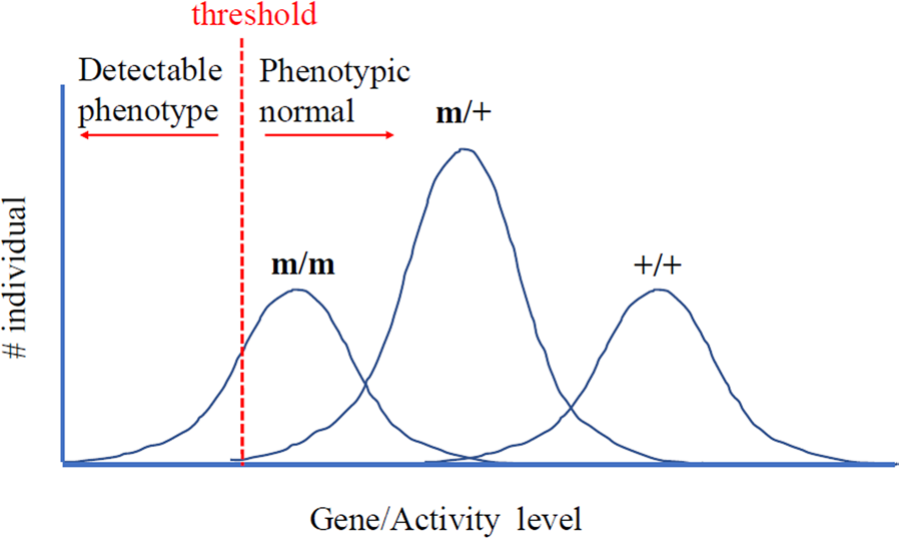
Threshold determines penetrance. We use a hypothetical case to illustrate the conceptual relationship between threshold and penetrance. Consider a hypomorphic allele m of a gene X. The X-axis is the level or activity of X. The Y-axis is the number of individuals with a particular [X]. Due to many factors, the [X] displays a distribution curve. A hypothetical threshold for [X] is drawn. If [X] falls below the threshold, the individual will exhibit the clinical phenotype. If [X] is above the threshold, the individuals will be phenotypically normal. In this hypothetical example, the ***m*** variant is recessive, because all ***m/ + ***individuals will be above threshold. The ***m*** variant is incompletely penetrant, because only a fraction of ***m/m*** individuals will be below threshold and exhibit phenotypes. If the threshold shifts to the right, the penetrance will become higher, i.e., more individuals are below threshold. If the threshold shifts to the left, then less individuals are below threshold, hence a lower penetrance

The concept of threshold to explain variable penetrance and expressivity was originally proposed by Richard Goldschmidt in 1927 (see [51] and in recent reviews [6, 49, 52–54]). Indeed, the examples described above ultimately cause a quantitative reduction of a certain critical molecular component. Hence, a phenotype may develop if the level or activity of a critical factor falls below a threshold. As all individuals with the critical factor below the threshold level develop the phenotype, the threshold determines the penetrance (Fig. 2). Shifting the threshold will thus change the penetrance, i.e., the fraction of individuals to exhibit the phenotype. Moreover, different tissues can be differentially affected by a broadly expressed factor, causing pleiotropy. For example, the expression level of a critical factor may vary in different tissues and some tissues may be below the threshold and hence exhibit a phenotype. Furthermore, the threshold may vary in different tissues (e.g., variation in the level of a cofactor), causing further tissue-specific variation in phenotypes. In summary, a threshold effect may turn a continuous quantitative difference of a critical factor into qualitatively different phenotypic outputs, and may explain the context dependence of tissue-specificity and temporal-dependence. The threshold effect describes an input–output relationship that exhibits a sharp non-linear, non-deterministic transition when a certain level of input is reached.

### Ultrasensitivity as a molecular mechanism for the threshold effect

**Fig. 3:**
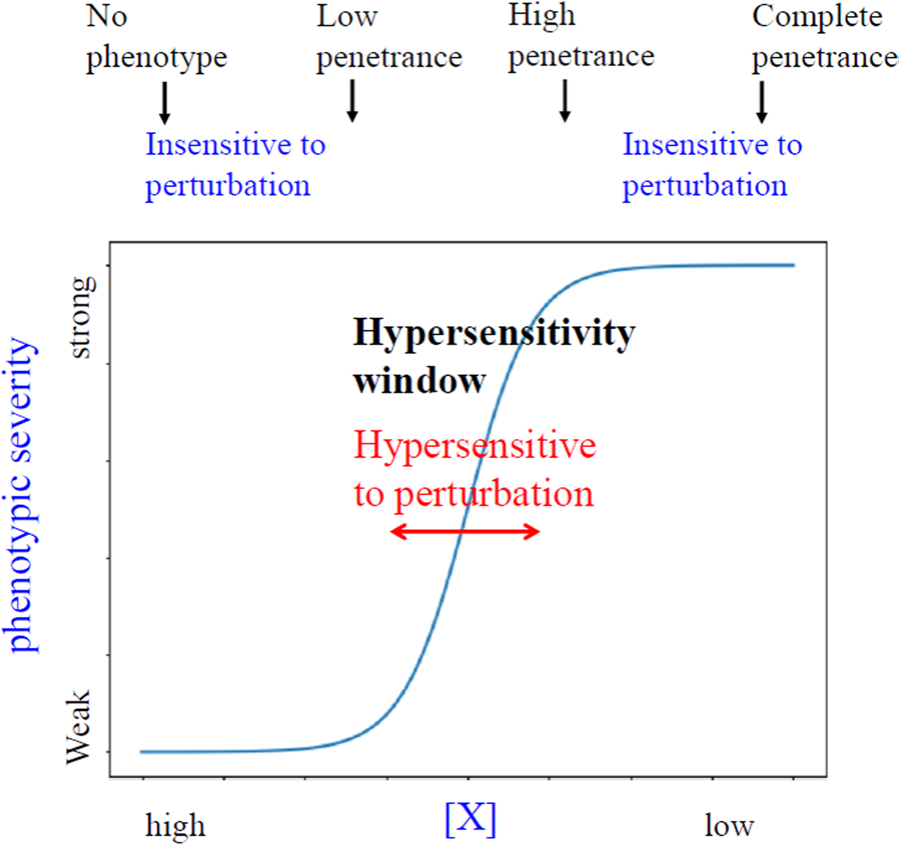
A sigmoidal curve illustrates the threshold effect. The phenotypic severity (Y-axis) is plotted with the level or activity of factor X (denoted [X]) in the X-axis. In a sigmoidal curve of such “dose–response” curve, when [X] is at its normal dosage (high), the system is at its normal (wildtype) state. When [X] is reduced due to a low level, e.g., due to a null mutation in X, the system exhibits the mutant phenotype with complete penetrance. At these two states, small perturbations in [X] do not cause any changes in the phenotypic output. However, at near the inflection point of the curve, small changes in [X] will cause large changes in the phenotypic outcome. Therefore, the system is ultrasensitive to changes in [X]

**Fig. 4:**
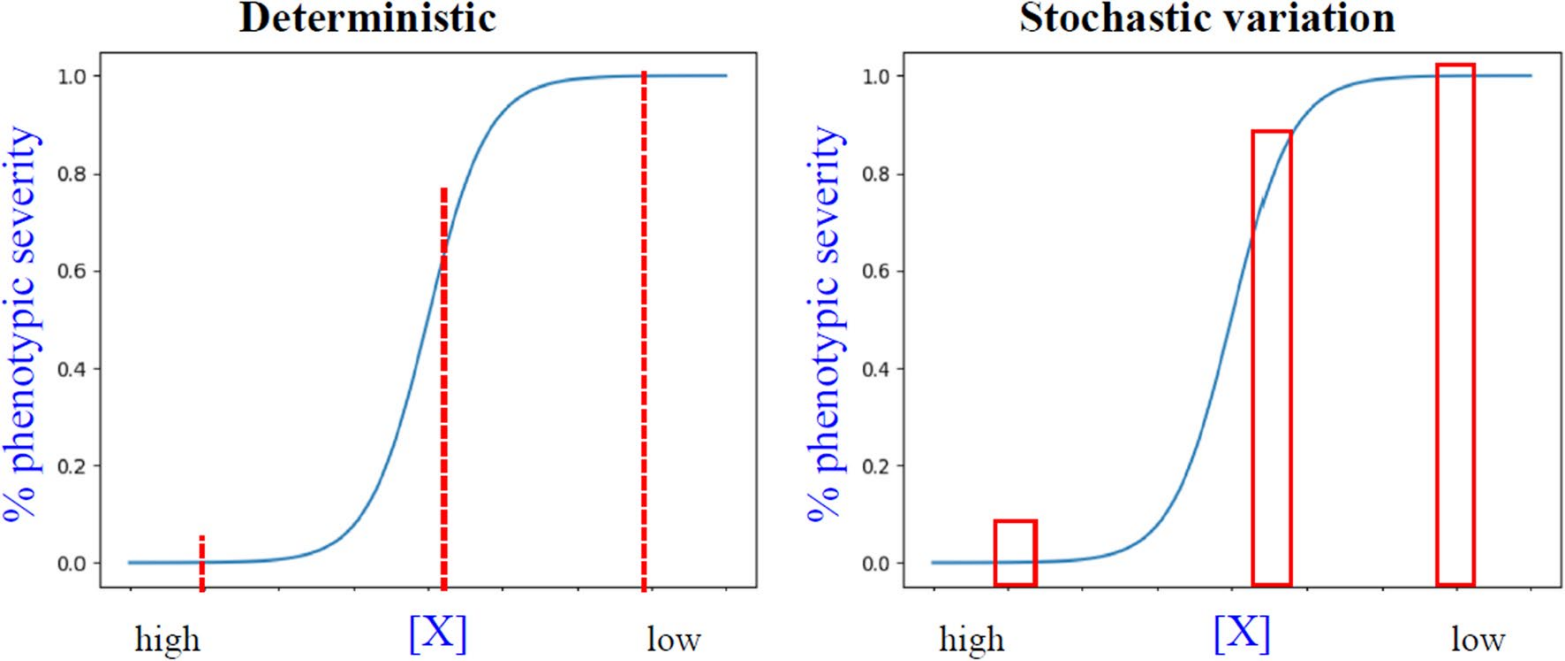
Stochastic variation in input causes heterogeneous output. (Left) A fixed [X] would generate a fixed output in phenotypic severity. (Right) Stochastic fluctuation in [X] generates different [X] in different individuals or cells, thereby generating a range of phenotypic output in different individuals

What constitutes a threshold at the molecular level? The sigmoidal curve is a good representation of a threshold effect (Fig. 3). The output (phenotypic manifestation) rises sharply over a narrow range of concentration or activity of a key component ([X]). In such a system, perturbations of [X] within the range at the two ends of the curve do not cause any change in phenotype. The system exhibits a wild-type phenotype at one end, and mutant phenotype with complete penetrance at the other end. When [X] is reduced to near the inflection point of the sigmoidal curve, a small perturbation of input ([X]) can cause a significant change in output. Within this window of [X], the system is ultrasensitive to perturbations on [X]. A fixed [X] would produce a fixed output in phenotypic severity. However, if the input [X] is stochastically variable in different individuals, the system will then exhibit heterogeneity in phenotypic output (Fig. 4). This changes the input–output relationship from deterministic to a stochastic, probabilistic relationship.

The slope of the sigmoidal curve captures the degree of ultrasensitivity, which may also be expressed in terms of a Hill coefficient [55]. The slope affects the range of phenotypic variation. A steep curve means a broad range of variation, i.e., more phenotypic heterogeneity, over a small change in input. The range of phenotypic heterogeneity is highest when the system is near the inflection point.

**Fig. 5:**
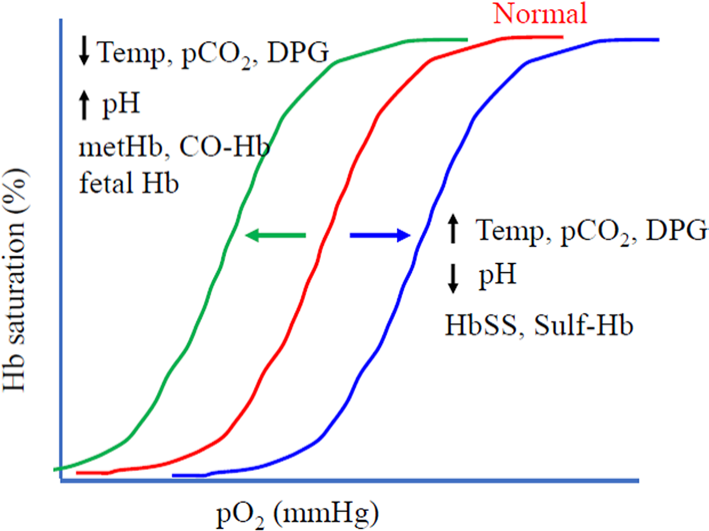
Threshold can shift. The hemoglobin-oxygen binding is a classic example of positive cooperativity and exhibits a sigmoidal curve (red curve). The curve can be shifted rightward (blue curve), representing reduced affinity for oxygen, by elevated temperature, CO_2_**,** and 2,3-diphosphoglycerate (DPG), and reduced pH, resulting in increased threshold. Sickle cell hemoglobin (HbSS) and sulfhemoglobin (Sulf-Hb) have reduced affinity to oxygen, so exhibits right-shifted curve. The curve can be shifted leftward (green curve), representing increased affinity for oxygen, by reduced temperature, CO_2_**,** and DPG, and elevated pH**.** Methemoglobinemia (metHb), fetal hemoglobin and CO-bound Hb (CO-Hb) have higher affinity to oxygen, so exhibit leftward curve. These examples demonstrate that the threshold can be shifted by environmental or physiological factors, as well as by changes in the protein structure. Adapted from [128]

The threshold effect can be context dependent. The sigmoidal curve can shift to right or left due to changes in environmental conditions (e.g., pH, temperature), or tissue-specific gene expression, thereby changing the threshold (Fig. 5). Therefore, these ultrasensitive responses can be non-linearly sensitive to changes in environmental conditions or cellular context, providing an explanation for pleiotropy. The threshold effect can also be time dependent. There can be a critical time window for a threshold decision [56]. This may be due to the transient expression of a factor to form a particular gene regulatory network (GRN) topology within a narrow time window.

Note that the sigmoidal curve refers only to the change in the concentration/activity of X. There could be other factors (Y, Z, etc.) in the regulatory network. The relationship of phenotypic output versus changes in these other factors can be described by different response curves, which may or may not exhibit the ultrasensitivity response. Changes in factor Y alone may not cause a phenotype, but in combination with a variant in X may enhance or suppress the X phenotype. This is the basis of genetic epistasis and the key to understanding complex diseases.

Classical examples of sigmoidal curves derive from allosteric positive cooperativity, e.g., multiple binding sites that have direct or indirect interactions affecting enzymatic activity. For example, enzymes that have multiple binding sites where the binding of the first ligand increased the binding of the second ligand [57, 58]. In addition, many ultrasensitive response motifs (URMs) have been found in regulatory networks [55, 59–61], either at the transcriptional or protein interaction level, and can exhibit a similar property, i.e., sharp transition over a narrow range of concentrations. These include positive cooperative binding, negative cooperative binding, homo-multimerization, multistep signaling, molecular titration (by stoichiometric inhibitor), covalent modification cycle (e.g., phosphorylation and dephosphorylation cycle), positive feedback, and reciprocal regulation [57, 59, 62–73]. There are thus multiple ways to generate ultrasensitivity. Therefore, ultrasensitivity provides a molecular mechanism for the threshold effect. Importantly, many of these URMs are frequently found in genetic regulatory networks.

### Three conditions for ultrasensitivity-mediated phenotypic heterogeneity

**Fig. 6:**
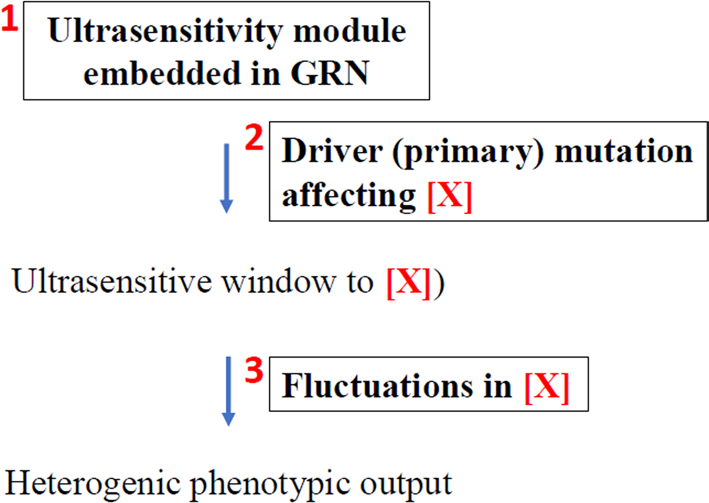
The three conditions for threshold effect. First, an ultrasensitivity module need to be embedded in the gene regulatory network. The system is normally in a stable (homeostatic) state, i.e., the wildtype state, and is robust to small fluctuations. Second, the occurrence of a primary (driver) mutation shifts the system to a state that is ultrasensitive to changes in the concentration/activity of factor X ([X]). If [X] does not fluctuate, then the system would have a fixed [X] and a fixed outcome. Third, fluctuations in [X] would cause the system to produce heterogeneous phenotypic outputs

We propose that phenotypic heterogeneity in genetic diseases requires three conditions (Fig. 6). First, the regulatory network needs to have an embedded URM. Although it has the potential for ultrasensitivity, the system is robust to small fluctuations and normally in a stable (homeostatic) state, i.e., the wild-type state. Second, a primary (driver) variant reduced the concentration/activity of factor [X] to a point near the inflection point so the system shifts from the stable state to an ultrasensitive state that responds to the changes in [X]. The driver mutation could be in X itself, e.g., a partial loss that reduces [X] to the ultrasensitive window. The driver mutation could also be in a different gene but affect [X]. The system is now poised for ultrasensitive response. However, if [X] does not fluctuate, then the system would have a fixed [X] and a fixed outcome. Third, fluctuation in [X] might cause the ultrasensitive system to respond with heterogeneous phenotypic outputs. Changes in [X] can be caused by stochastic variation in [X], second site mutation (in modifier genes) causing variation in [X], or non-genetic (stochastic or environmental) variation in modifier genes causing variation in [X]. In principle, modifier genes may act by changing [X] itself, the magnitude of variation in [X], or the threshold to achieve the same effect of phenotypic heterogeneity.

**Fig. 7:**
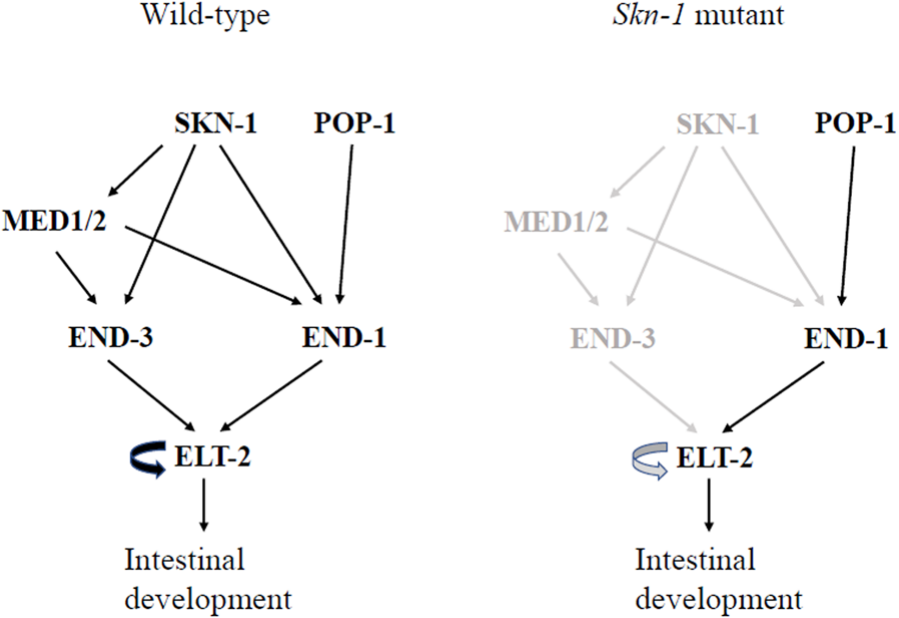
ELT-2 regulation as an example of the three requirements for phenotypic heterogeneity due to ultrasensitivity mediated threshold response. In *Skn-1* mutants, the ELT-2 positive feedback is weakened due to absence of END-3 and reduced END-1. The ELT-2 level may fall below its functional threshold in some individuals, causing a failure in in *C. elegans* intestinal development. Modified from [56]

One example is intestinal development of *C. elegans*, regulated by SKN-1 which activates MED-1/2 and END-3. The three in turn activate ELT-2 [56, 74] (Fig. 7). First, ELT-2 is regulated by a positive feedback loop, which is a potential URM. Second, the *skn-1* mutation constitutes the primary mutation that shifts the system to the ultrasensitive window. In a *skn-1* null mutant, MED-1/2 and END-3 expression is lost and END-1 expression is reduced and now only dependent on POP-1. The reduced END-1 may cause the ELT-2 positive feedback loop to be ultrasensitive to the level/activity of ELT-2. Third, stochastic fluctuations in END-1 levels cause variation in the ELT-2 level. In some cells, the reduced END-1 induces a low level of ELT-2, below the threshold to activate positive feedback, leading to a failure in intestinal development. In some cells, the END-1 level induces ELT-2 above the threshold for positive feedback and hence normal intestinal development. This is a plausible explanation for the incomplete penetrance of the *skn-1* mutant phenotype.

Note that all three conditions need to be met for phenotypic heterogeneity to occur. Normal individuals are robust to extrinsic and intrinsic perturbations. Simply having an embedded URM or having quantitative variations is not sufficient to cause phenotypic heterogeneity. Only when the system is within the ultrasensitive window, due to a primary mutation, will small fluctuation become a key component that can cause strong variability in the phenotypic output.

### Threshold effects at the organ level

The above analysis is based on regulatory networks at the cellular level. Do similar principles apply for interactions among cells at the tissue/organ level? If we substitute [X] from the concentration/activity of a molecule X with the activity of a cell type [X] and plot against the percent dysfunction of the organ, are there conditions that can generate a sigmoidal curve (ultrasensitive response)? Is organ failure a linear or gradual functional decline due to progressive loss of functional units, or due to a sharp transition due to a threshold response?

There are suggestions of thresholds for organ failure. One indicator of kidney function is the excretion rate of sodium. If the sodium excretion rate is plotted against the concentration of a diuretic, the dose–response curve is sigmoidal [75]. This curve in chronic kidney disease (CKD) patients is shifted so that the inflection point is at a higher plasma level of diuretic, meaning that CKD patients respond to diuretic drugs at a higher threshold concentration. For the lung, the static volume-pressure curve is sigmoidal [76]. For heart failure, there is a threshold for left ventricular ejection fraction that may be useful for clinical classification [77]. These examples of sigmoidal curves in input–output responses measured at the organ level suggest that there are threshold effects at the organ level. Whether these organ level phenomena can be understood at the level of cell–cell interactions similar to ultrasensitivity regulatory motifs remains to be studied. Inter-cellular and inter-organ communications in heart failure are being studied [78, 79] and hopefully will provide more quantitative insights.

Neurodegenerative diseases may represent examples of threshold effects at the brain level. In the *Drosophila drop-dead* mutant, the brain shows extensive degeneration long before the sudden death of the flies [80], suggesting that there is a threshold in neuronal dysfunction. In Parkinson’s disease (PD), the decline in the number of dopaminergic neurons over years is a sigmoidal curve and a 70% loss of dopaminergic neurons is typically observed when PD motor symptoms occur, suggesting a threshold effect [81]. In contrast, the brainstem, and peripheral, autonomic and enteric nervous systems show symptoms when only 20–30% of dopaminergic synapses are lost in PD patients. The difference in threshold between the two systems has been suggested to be due to differential sensitivity to α-synuclein aggregation. The less connected brainstem, peripheral, autonomic and enteric nervous system may have less “functional reserve” and therefore would be more sensitive to α-synuclein aggregation, while the highly connected midbrain is more resilient, and hence exhibits a higher threshold for dopaminergic neuron loss [81]. This example not only suggests a threshold effect, but also suggests that the threshold can have context-dependent differences.

Quorum sensing is an example of cell–cell signaling that exhibits ultrasensitive responses in a population of cells [82, 83]. Cells secrete a molecule, whose concentration is used as an index for cell density. A change in cell behavior in the population is triggered only when the quorum sensing molecule reaches a threshold concentration. Bacterial quorum sensing commonly uses positive feedback as its ultrasensitivity motif [84]. The threshold effect creates phenotypic heterogeneity in the bacterial population [85]. Although quorum sensing is often studied in microbial populations, it has been demonstrated in hair follicle regeneration, which is induced only when a threshold density of injury occurs [86]. Potentially, a similar mechanism also applies to cell interactions at the organ level.

Cell–cell interactions have been predicted by multiple approaches based on transcriptomics and protein–protein interactions [87]. Jung et al. [88] used tissue-specific single cell transcriptomics to establish cell–cell interactome. Among the cell–cell interactions, they identified positive feedback loops between cell types. Positive feedback loops specific to pathological conditions can be identified, and perturbation of these disease-specific ligand-receptor pairs can be simulated to identify those with strong effects. Whether these potential ultrasensitivity motifs actually exhibit ultrasensitivity in the context-of-interest needs to be experimentally tested.

### Pleiotropy and edgetic mutations

**Fig. 8:**
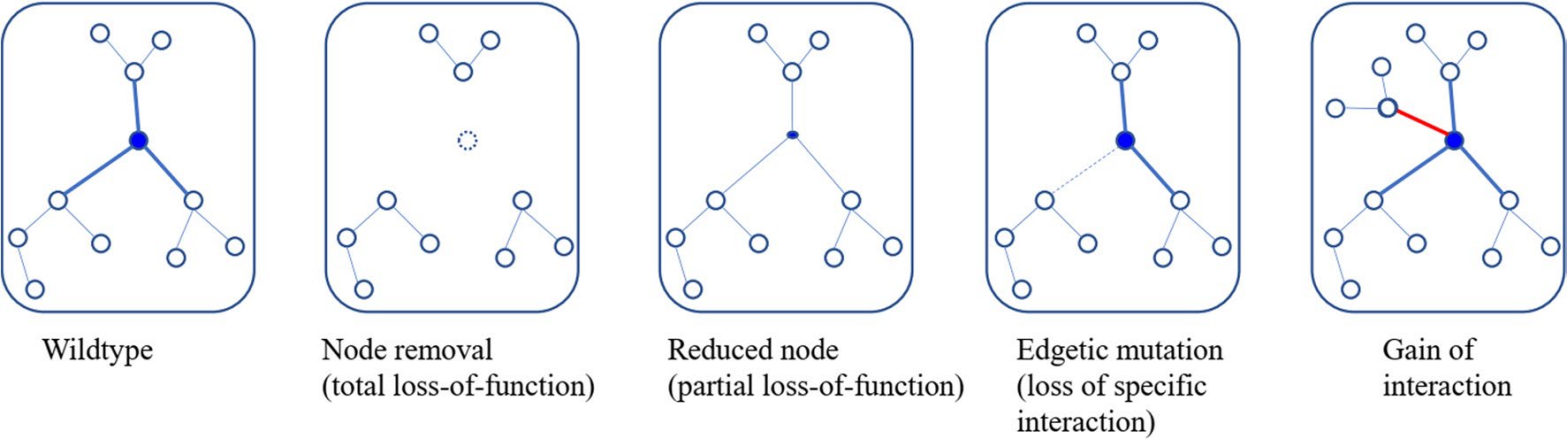
Protein interactions affected by different types of mutations. The dots represent proteins and lines (edges) connecting dots represent their interactions. Different types of mutation may affect the node or the edges. Modified from [89]

For simplicity, the above analysis considers only loss-of-function mutations (loss or reduction of gene/protein level). These can be total or partial loss-of-function mutations. However, other types of mutations are potentially important as well. Consider a network of protein interactions (Fig. 8). Each protein is a node that is connected to interacting proteins. The interactions are described as edges. Proteins can have multiple structural domains that interact with different proteins. These binding proteins may bind to independent sites, compete for the same site, or bind to distinct sites but they can interact with a protein bound to another site or affect other protein interactions. Some mutations may affect only one such binding interface, thereby affecting interaction with specific proteins, while leaving other interactions intact or only slightly affected. Such mutations are called edgetic, because they affect the edge, not the node, in the protein interaction network [89, 90]. Edgetic mutations can also be gain-of-function mutations by creating new interactions.

Since an edgetic mutation may affect only one edge, it may cause only a subset of phenotypes associated with loss of function of the node protein. This provides one possible explanation for heterogeneity in pleiotropy. If the edges have unequal strength in their interactions, then one mutation that reduces the node level by 50% can affect edges differentially, i.e., some edge/interaction is affected more strongly. This can also contribute to the differential phenotypic heterogeneity in different tissues. Because a mutation may affect different contexts differently, it can be deleterious in some contexts, but provide positive fitness effect in other contexts and therefore be maintained in evolutionary selection. The TP53 oncogene is one example of such antagonistic pleiotropy [91, 92]. Another example is the antagonistic pleiotropy proposed to explain aging, stating that a pleiotropic mutation may be positively selected for early-life function, but leads to negative fitness in late-life [93]. Antagonistic pleiotropy is common [93] and is often buffered by compensatory mutations in evolution [94]. The effect of the compensatory mutations may be context-dependent.

High resolution (structurally resolved) protein interaction networks can predict the interacting sites between interacting proteins [95]. Protein 3D structures can now be predicted efficiently by AI [96–98]. High resolution protein interaction databases are being established that permit the mapping of mis-sense variants to the protein of a known disease gene and may help to establish if a specific protein interaction is affected [99]. Establishment of tissue-specific functional interaction networks would help to identify tissue-specific interactions [100] accounting for pleiotropy. Studying patients who display pleiotropy may help identify edgetic mutations and identify the tissue-specific interaction protein partner.

### Research strategy based on the threshold model

Phenotypic heterogeneity is often regarded as nuisance and noise in research. However, we argue that it is valuable in pointing out potential molecular mechanisms of diseases. Each of the three conditions proposed for phenotypic heterogeneity would provide new insights into disease mechanisms. We will attempt to give some suggestions for research strategies based on these three conditions. First, we need to identify the primary mutation and modifier factors (enhancer and suppressor mutations, environmental factors), which need to be functionally validated for their causal relationship. Second, we need to identify the URM in the regulatory network that may account for heterogeneity. Third, we need to find ways to nudge the system from an ultrasensitive state back to a stable healthy state. These points are separately discussed below.

### Identification and functional validation of the primary and modifier mutations for a disease

The threshold model leads to some suggestions on how to choose patients to study a particular disease. It is best to focus on familial cases, as these indicate sufficiently high penetrance in order for them to be recognized as familial cases by clinicians. Although the small sample size may give low statistical power, the possibility of comparing genomic information among family members can help to reduce the number of candidate genetic variants. Familial cases with phenotypic heterogeneity in carriers are suggestive that the system is within an ultrasensitive window. One should look for extreme cases (e.g., very early or very late onset, very strong or very weak phenotype) within such families, because these individuals may harbor strong modifier mutations. Carriers that show a very weak phenotype or very late onset may harbor protective alleles. Pleiotropic phenotypes may be useful to identify edgetic mutations that affect tissue-specific interactions with interacting proteins [89, 90].

Animal models of disease are often used to screen or analyze modifier mutations that can enhance or suppress the original mutant phenotype. From these, the mechanistic chain can then be established. This is where model organisms are most advantageous in providing mechanistic insight for human diseases, since many disease genes are evolutionarily conserved [101]. It is important to keep in mind that phenotypes can be highly dependent on the genetic background of an animal [25], suggesting context dependence of ultrasensitivity. The modifier mutation screen should be performed in a sensitized genetic background (i.e., within the ultrasensitivity window) and in tissues with regulatory network topology similar to that of human disease tissue. This may be experimentally identified by observing which tissue mimics the human disease phenotype. Because the regulatory network may vary in different genetic backgrounds, it may be useful to screen/test in multiple genetic backgrounds and tissues. Different genetic backgrounds may identify different modifiers. For economic reasons, this is more feasible in model organisms such as yeast, *C. elegans* and *Drosophila* [102].

The identified candidate mutations need to be functionally tested to assess whether they are causal to the disease phenotypes. Often this is done by creating or expressing a genetic variant in an animal model or in cell lines [102]. Model organisms are usually designed to be homogeneous in genetic background. Therefore, each animal model or cell line may represent only a fraction of the human heterogeneous genetic background. Additionally, the culture conditions for cell lines or animal models is usually selected to minimize environmental variations, which is different from the variable environmental conditions that humans encounter. This may be the reason why so many drugs developed using cells or animal models failed when tested on humans. The context-dependence of phenotypic heterogeneity suggests that different tissues or organs are different in their regulatory network topology. These regulatory networks may not be conserved in all tissues between humans and the model animals. Therefore, an animal disease model may recapitulate only part of the phenotypes of a human disease. This does not diminish the value of animal disease models, but cautions on the context dependency. One can try to focus on the phenotype that most closely mimics the human phenotype, suggesting that such tissue has similar network topology and is within the ultrasensitive window. If the conditions for ultrasensitivity are clear, they may be used in patient stratification, which would greatly facilitate drug testing.

### Identifying URMs for a disease

There are many ways to infer regulatory networks, e.g., based on coexpression, sequence motifs, chromatin immunoprecipitation, orthology, literature and protein–protein interactions for transcriptional regulatory relationships [103]. Recent advances in single cell transcriptomics have helped to construct cell-type-specific regulatory networks and compare them between healthy and disease states (e.g., [104, 105]. Regulatory network motifs with potential ultrasensitive behavior can be identified by simulation and searching through the entire space of potential motifs and through parameter space [106].

Even without using these sophisticated analyses, many ultrasensitive motifs can be easily identified. For example, a positive feedback effect can be identified by simple transcriptomic analyses of a mutant tissue to determine whether the gene in question affects its own expression. Molecular titration by stoichiometric inhibitors is often used to set a threshold, and is exemplified by corepressors to receptors and transcription factors [107, 108] and antagonists to signaling ligands, e.g., Chordin and its fly homolog Sog as antagonist to BMP; [109, 110]. Sex determination in Drosophila is very sensitive to a threshold set by the X:autosome ratio which is determined by binding between the autosomal bHLH repressors Deadpan and the X-chromosomal SisA and SisB bHLH proteins to titrate out SisA and SisB for binding with the maternal Daughterless, a bHLH transcriptional activator of the Sxl gene [111]. Positive cooperative binding is often seen with the binding of transcription factors (TFs) to target genes with clustered binding sites in the promoter region, e.g., Bicoid activates target gene hunchback expression in a sharp domain through positive cooperative binding to its binding sites in the hunchback promoter [112]. Therefore, clustering of TF binding sites is suggestive of cooperative binding. Homo-multimerization is often seen in transcription factors and receptors, e.g., ligand-dependent dimerization of receptor tyrosine kinases that critically affects their activity (e.g., [113]). For covalent modification cycle with zero-order ultrasensitivity, i.e., operating near substrate saturation, there are many examples of protein phosphorylation, acetylation and methylation (e.g., [114]) as candidates for ultrasensitivity.

However, just knowing regulatory topology does not guarantee ultrasensitivity. Notably, these motifs exhibit ultrasensitivity only within certain parameter ranges. For example, for a single-site phosphorylation-dephosphorylation cycle, ultrasensitivity occurs when the catalyzing enzymes is operating near saturation, i.e., substrate concentration is high (zero-order, i.e., reaction kinetics is independent of substrate concentration), and with no product inhibition or sequestration, i.e., the enzyme is quickly released from its substrate after its reaction [67, 115, 116]. Multisite phosphorylation exhibits ultrasensitivity when there is positive cooperativity, i.e., phosphorylation of the first residue accelerates the subsequent phosphorylation steps [117]. However, ultrasensitivity can also occur even in the absence of positive cooperativity in a multisite system [118]. Therefore, in addition to identifying the potential URMs, one should also examine whether these parameter requirements are also met in the pathological condition. Experimental validation in model organisms or cells are needed.

### Manipulating thresholds for disease prevention or therapy

Robustness, i.e., insensitivity to variations, is a property opposite of heterogeneity. To achieve robustness, the system is normally buffered against perturbations [119]. The buffering can be due to network properties, e.g., negative feedback, for homeostasis. Buffering can also be due to protein chaperones, e.g., Hsp90 [120–123]. The system is normally at a stable healthy state, i.e., above the threshold, and not ultrasensitive to perturbation. The disease state is caused by reducing the weak spot (Achilles heel, or tipping point) to shift the system to an ultrasensitive window. Conversely, by knowing the ultrasensitive motif, we can try to manipulate the weak spot and shift the system from an ultrasensitive state to a stable state. Identifying the critical factor and its position in the regulatory network is important to find ways to manipulate the threshold. Another approach is screening for second site dominant suppressor mutations, as these would be dosage-sensitive and may act to shift the system back to a stable phenotypically normal state.

In the above example of the *skn* mutant phenotype in *C. elegans* intestinal differentiation, ELT-2 is the critical factor involved in a positive feedback loop (Fig. 7). Potentially finding ways to increase ELT-2 expression, activity, or stability may shift the system to a stable state with normal intestinal development even in a *skn* mutant background. The molecular titration URM can be modulated. If an active factor A is bound by a protein B, which renders the AB dimer inactive, this protein sequestration system can exhibit ultrasensitivity behavior [124]. The threshold and degree of ultrasensitivity depend on the concentration of the inhibitor B [124]. Therefore, the level of B can be manipulated to adjust the ultrasensitivity and potentially push the system into a stable state. Posttranslational modification can also be used to modulate system output [125]. These examples show that if we understand the molecular mechanism for ultrasensitivity, we may be able to manipulate the system to push it back to a stable healthy state.

Drug development usually tries to identify a druggable target acting within a regulatory chain, and then try to modulate its level or activity. However, how to choose which of the multiple potential targets along the regulatory chain is difficult. In contrast to traditional approaches in identifying druggable targets, our model suggests that it is important to know the regulatory network topology and identify the ultrasensitive response motif, which would be the tipping point that is most sensitive to perturbation. The tipping point would be the drug target. A gentle nudge on the tipping point may be sufficient to shift the system. Because a smaller drug dosage may be effective, side effects can be reduced. This is of course a tall order that is more easily said than done. Much experimental effort in testing the predictions and showing validation is needed.

In summary, based on the threshold model, we make the following suggestions on research strategies dealing with phenotypic heterogeneity in human diseases.

1. Identify the primary mutation and modifier factors for a disease
  Focus on familial cases, extreme cases (enhancers and suppressors) in carriers within family
  Pleiotropy may help to identify edgetic mutations
2. Functional validations of causal relationship
  Remember context dependence
3. Look for ultrasensitive response motifs in the regulatory network
  Need for experimental validation
4. Find ways to nudge the system from hypersensitive state to stable state
  Screening for dominant suppressor mutations
  Identify the tipping point as the target for manipulation.

## Conclusions

We have proposed that the ultrasensitivity response is the molecular basis for the threshold effect which can be an underlying principle for the phenotypic heterogeneity in human genetic diseases. It is noted that “the majority of the research in model organisms aims to minimize background effects rather than understanding it” [126]. We argue that the phenotypic heterogeneity should not be treated as noise in the system but that it can reveal the potential molecular mechanisms underlying these phenotypes. We should take advantage of pleiotropy and background differences in human and animal models to identify context-specific regulatory networks associated with disease. Conceptually, if we can understand the network topology and the parameters of a system, we can predict the behavior of the system, especially whether it can exhibit ultrasensitivity under certain parameters, and in so doing may find ways to gently nudge the system to stability. The understanding of network behavior is important. Context is also important. In reality, this is quite difficult and needs to be experimentally tested, which may be a daunting effort. The combinations of ‘omics data, with better spatial and temporal resolution from patients and animals models, experimental testing of quantitative variables in animal models, and mathematical simulations are needed. However, the potential payoff is great. It would be possible to develop gentler and more effective forms of prevention and therapy, and avoid side effects.

We have discussed heterogeneity with respect to diseases. The assumption is that the body is normally in a homeostatic state, and only upon certain disease conditions (e.g., the driver mutation) is it shifted to a state that is sensitive to perturbations into a disease state. However, in some situations, the organism seems to be deliberately poised at an ultrasensitive state that responds to small intrinsic or extrinsic fluctuations with large differences in phenotypic output, so that individuals respond in dichotomous ways to a common set of circumstances. For example, not all cherry trees blossom at the same time, and not all flowers on the same tree blossom at the same time. This “bet-hedging” strategy may have evolutionary value to a species [85, 127]. The threshold model may also be useful in this broader realm of phenotypic heterogeneity.

## Data Availability

Not applicable.

## Abbreviation

URM: Ultrasensitive response motif
SMA: Spinal muscular atrophy
OXPHOS: Oxidative phosphorylation
GRN: Gene regulatory network
CKD: Chronic kidney disease
PD: Parkinson’s disease

## Glossary

Penetrance: The percentage of individuals carrying a particular genotype (e.g., a mutation) that exhibits certain detectable phenotypes (clinical manifestation). Complete penetrance means 100% genotype–phenotype correlation.
Expressivity: The degree of phenotypic severity in an individual who exhibits detectable mutant phenotype.
Pleiotropy: A gene, when mutated, is linked to multiple phenotypic traits, often in multiple tissues or organs.
Stochasticity: Non-deterministic variations that lack predictability or order.
Threshold: A hypothetical value of functional requirement for the concentration or activity of a molecule X (denoted as [X]). If [X] falls below such value, phenotypic defects will manifest.
Ultrasensitivity: In an input–output relationship, a small change in the input causes a large, non-linear, change in the output, resulting in a switch-like or bistable behavior.
Ultrasensitive response motif (URM): A regulatory motif that can give an ultrasensitive response under appropriate parameters.
Edgetic mutation: Mutation affecting a protein not in the production of level of the protein itself (node), but specifically affects one (or a few) of the protein interactions (edges) in the protein interaction network.
